# Genistein Supplementation and Bone Health in Breast Cancer in Rats

**DOI:** 10.3390/nu16060912

**Published:** 2024-03-21

**Authors:** Dorota Skrajnowska, Wojciech Bielecki, Arkadiusz Szterk, Karol Ofiara, Barbara Bobrowska-Korczak

**Affiliations:** 1 Department of Toxicology and Food Science, Faculty of Pharmacy, Medical University of Warsaw, Banacha 1 Street, 02-091 Warsaw, Poland; 2 Department of Pathology and Veterinary Diagnostics, Institute of Veterinary Medicine, Warsaw University of Live Sciences, Nowoursynowska 159c Street, 02-787 Warsaw, Poland; 3 Center for Translational Medicine, Warsaw University of Life Sciences, Nowoursynowska 100, 02-797 Warsaw, Poland; 4 ASLab Science, Fort Służew 1/9, 02-787 Warsaw, Poland

**Keywords:** bones, mineral composition, cancer, genistein

## Abstract

The aim of our study was to analyse the effect of supplementation with various forms of genistein (nano-, micro-, and macro-) on the mineral status of rat femurs in conditions of DMBA-induced mammary gland neoplasia. Thirty-two 30-day-old Sprague Dawley rats were used in the study. The rats were divided into four experimental groups: a control group (without supplementation) and groups supplemented with nanosized (92 ± 41 nm), microsized (587 ± 83 nm), and macrosized genistein. Micromorphometric and histological examination of the rat femurs were performed, as well as analysis of the weight and mineral composition (17 elements). Quadrupole ICP-MS was used for analysis of all trace elements. Supplementation with genistein (nano-, micro-, and macro-) was shown to cause changes in the mineral composition of the bones. In the rats receiving nanogenistein, disintegration of the bone tissue was observed. The femurs of these animals had higher content of calcium (by nearly 300%) and potassium (by 25%) than the other groups, while the level of magnesium was about 22% lower. In the case of microelements, there were increases in copper (by 67%), boron (48%), manganese (13%), and nickel (100%), and a 16% decrease in strontium compared to the bones of rats without genistein supplementation. Changes in micromorphometric parameters, resulting in increased bone fragility, were observed. Administration of genistein was found to have an effect on the amount of trace elements in the bone tissue of rats with breast cancer.

## 1. Introduction

In 2020, about 2.3 million cases of breast cancer were diagnosed in women around the world. By 2040, this number is projected to increase by more than 40%, and the number of deaths is expected to increase from 685,000 in 2020 to one million in 2040 [1]. In the search for compounds with anticancer activity, a great deal of attention is focused on genistein. Genistein, a natural isoflavone (5,7-dihydroxy-3-(4-hydroxyphenyl)-4H-1-benzopyran-4-one) present in soya and other legume plants, exerts beneficial biologic effects, including anti-inflammatory [2], antioxidant [3], and anti-tumour [4,5,6] effects, as well as protecting bones and cartilage [7]. Genistein can exhibit anti-tumour activity in part by inducing apoptosis, affecting the cell cycle, inhibiting angiogenesis, and exhibiting antiproliferative activity. Although many studies have shown that intake of genistein can prevent the development of breast cancer [8], the effect of genistein on active breast cancer cases remains controversial [9]. The role of genistein in breast cancer is ambiguous and determined by multiple factors, including dose- and age-dependent biological effects, the ratio of alpha and beta oestrogen receptors, gene mutations, individual differences in metabolism, and finally the possibility of action through various metabolic pathways [10,11]. One of the best-known properties of genistein is its oestrogenic activity. Genistein is a relatively strong agonist of the oestrogen receptor beta isoform (ERβ). Even nanomolar serum concentrations of free unbound genistein, which can be attained through intake of soy isoflavones, e.g., in a typical Asian diet, allow it to be bound to this receptor and are an order of magnitude lower than the affinity of genistein for the oestrogen receptor alpha isoform (ERα). Genistein shows similar activity to oestrogen in protecting against osteoporosis and degenerative joint disease [12,13,14]. Many studies indicate that this highly beneficial role of genistein is linked to inhibition of IL-6 production (even at physiological concentrations) and increased production of osteoprotegerin in human osteoblast-derived cell lines [15,16,17,18]. Genistein can potentially also enhance bone metabolism by positively influencing vascular eNOS (endothelial NOS), an enzyme that mediates the osteogenic effect of oestrogen on osteoblasts. The inhibitory effect of genistein on osteoclasts in rats may be linked to inactivation of tyrosine kinase and activation of tyrosine phosphatase, and thus to increased concentrations of osteocalcin, indicative of osteoblast activity [7,19]. Another molecular mechanism by which genistein inhibits osteoclast formation may be linked to increased entry of calcium into the osteoclasts, ultimately inducing their apoptosis [20].

Many signalling pathways, such as MAPK (mitogen-activated protein kinases), NF-κB (nuclear factor kappa-light-chain-enhancer of activated B cells), and NRF2/HO-1 (nuclear factor erythroid 2-related factor 2/haem oxygenase 1), are modulated by genistein in protective mechanisms against bone and cartilage disease [19,21,22]. Genistein has been shown to induce the differentiation and maturation of multipotent bone marrow stem cells (BMSC) in various mechanisms, such as increasing the activity of p38MAPK-RUNX2 signalling pathways. RUNX2 is a key transcription factor of osteoblast differentiation, regulated by phosphorylation, which may be mediated by p38 MAP kinase and the NO/cGMP pathway (dose-dependent increase in the activity of NO synthase (NOS), NO formation, and cGMP production in BMSC cultures). Genistein can also reduce the formation of osteoclasts and bone resorption metabolism by blocking NF-κB signalling activity [23,24].

Epigenetic methylation of DNA by DNA methyltransferase (Dnmt) mediates gene expression and plays an important role in skeletal development [25]. Differentiation of mesenchymal stem cells to osteoblastic or non-osteoblastic progenitor cells is linked to the methylation rate of critical genes encoding BMP-2 (bone morphogenetic protein), a growth factor in the TGF-β (transforming growth factor β) protein superfamily. BMP-2 is actively involved in bone tissue metabolism, exhibits osteoinductive potential, and regulates the growth of cartilage, thus directly influencing osteogenesis. Classical alkaline phosphatase (ALP), also closely associated with osteoblasts, is regulated directly by BMP-2 or Wnt3a [26]. The Wnt pathway plays a number of important roles in embryogenesis, regulation of cell metabolism, proliferation and migration, tissue architecture, organogenesis, and homeostasis in adulthood [27]. For example, improper regulation and activation of these pathways has been shown to be linked to bone and cartilage diseases [28]. Genistein, curcumin, and quercetin show the ability to block the expression of target genes of the Wnt pathway, although their activity is also visible in earlier stages of this signalling pathway [27,29]. Research has shown that the effects of isoflavones can partially be ascribed to their effect on methylation of steroidogenic factor 1 [25,30]. Steroidogenic factor 1 (SF-1/Ad4BP/NR5A1) is a transcription factor involved in the development of gonad or adrenal tissue and differentiation of steroidogenic adult somatic stem cells [31,32,33]. Day et al. [34] found that the DNA methylation patterns changed in 8-week-old mice fed genistein in the amount of 300 mg/kg. Exposure of embryonic mouse tissues to genistein also modified the level of gene regulation [32]. Analysis of gene expression showed that the level of neuropeptide Y (NPY), a strong inhibitor of osteogenesis, was suppressed in a group receiving certain amounts of folic acid plus isoflavones (including genistein) through inhibition of factors such as runx2. NPY has also been shown to play a negative role in osteoporosis [33,35].

Epidemiological research has shown that intake of genistein can affect thyroid functions and thyroid hormone levels and indirectly affect the skeletal system [36]. Innate and juvenile hypothyroidism can delay skeletal development, whereas an excess of thyroid hormones can accelerate skeletal ageing, premature closure of growth plates, stimulation of osteoclastogenesis, and bone resorption [37,38]. Excessive levels of thyroid hormones in the body can cause osteoporosis because the excess hormones increase the excretion of calcium and phosphorus in the urine or faeces. Intake of genistein has been linked to high concentrations of TSH (thyroid-stimulating hormone), especially in women, which may be due to the inhibitory effect on the thyroid [39]. Genistein concentration in the urine, on the other hand, was correlated with a higher serum concentration of thyroxin (T4) in women, but not in men, and the authors ascribe the T4 level to the suppressive effect of genistein on the deiodination of T4 to T3 (triiodothyronine) [40]. Genistein is believed to reduce the level of thyroid hormones through competitive inhibition of the activity of thyroid peroxidase (TPO) and inhibit their binding to prealbumin, a transport protein for thyroxine [41,42,43]. Computer simulations have confirmed that genistein can bind to thyroid receptors, which was not confirmed in vitro [44]. Genistein has been reported to enhance transcription mediated by the triiodothyronine-liganded thyroid receptor [44]. It also inhibits aromatase and 17-β-hydroxysteroid dehydrogenase (17-β-HSD), which prevent the conversion of androgens to oestrogens, thereby reducing thyroxin-binding globulin (TBG) and the level of T4 [45,46].

To sum up, genistein models bone tissue in mechanisms reducing osteoclasts, reducing their survival or their formation, and at the same time increases the differentiation of primary osteoblasts by influencing oestrogen receptors and synthesis of nitric oxide [47,48].

The main problems in developing treatments based on genistein are its poor solubility in water, rapid metabolism, and rapid systemic elimination, resulting in low bioavailability and limited effectiveness. The results of many studies indicate that higher doses of genistein can induce a number of side effects by influencing numerous cellular pathways [13,49,50,51,52]. New forms of genistein are currently being created, such as nanoformulations [53]. In considering the possibility of genistein supplementation in nanoform, we need to test whether even relatively small doses will exert toxic effects. This is especially important as supplements sometimes contain more than 125 mg of pure genistein aglycone—more than five times the total average daily genistein intake for Chinese and Japanese adults [54,55,56]. Intake of the aglycone form of genistein can potentially lead to higher levels of unconjugated genistein in the blood due to better absorption in the stomach and increased uptake in the intestines. This is important from a biological perspective as unconjugated genistein has been shown to bind more strongly to oestrogen receptor β than its glucuronide metabolites, exhibiting the potential for a more oestrogenic effect on the bones [57]. The chemical structure of bioactive compounds is well-known to be closely linked to their biological activity [58]. The biological activity of genistein can be altered by structural modification, and even more so by major interference in the size of particles by reducing them to micro- or nanosized particles that can easily cross cell membranes and form adducts with important cell structures [59,60,61]. Given the positive effect of genistein in protection against osteoporosis, the question arises as to what effect isoflavone in nanoscale will have regarding the neoplastic process.

The aim of our study was to analyse the effect of supplementation with various forms of genistein (nano-, micro-, and classical) on the mineral status of rat femurs in conditions of mammary gland neoplasia. Despite numerous studies, the influence of genistein in nano- and microform on these issues in the stage of carcinogenesis is still not entirely clear. Reduction in materials at the nanoscale can lead to the development of new physical, chemical, and biological activities compared to macro compounds. It should be noted that there is still a lack of research in the literature in the field presented.

## 2. Materials and Methods

### 2.1. Laboratory Animals

Female Sprague Dawley rats (*n* = 32, 30 days) were obtained from the Animal Laboratory, Department of General and Experimental Pathology, Medical University of Warsaw. The study was approved by the Bioethics Committee at the Warsaw Medical University—document number—645/2018. All rats were provided with Labofeed H standard diet (Labofeed H, Żurawia 19, 89-240 Kcynia, Poland) and water ad libitum and housed in an environmentally controlled room at 22 °C with a 12 h light–dark cycle. The experiment lasted 100 days. After a 10-day period of adaptation to the experimental conditions, the animals were randomly assigned to groups of 8 individuals. The rats were divided into four experimental groups: control animals fed only the standard diet (without supplementation—0.4 mL water without supplements) and groups supplemented with nanosized (92 ± 41 nm; 0.1 mg/mL, i.e., 0.2 mg/kg bw), microsized (587 ± 83 nm, 0.1 mg/mL, i.e., 0.2 mg/kg bw), and microsized (classical) genistein (0.1 mg/mL, i.e., 0.2 mg/kg bw). The micro- and nanoforms of genistein were obtained by conventional grinding and homogenization. Preparation of genistein (micro- and nanoparticles) and parameters for evaluating the average particle size and zeta potential of the particles have been described in our previous work [62].

The rats were fed genistein supplements suspended in water (0.4 mL daily by gavage) from 40 days until 20 weeks of age. The level of genistein was selected based on human average daily consumption (extrapolating to the rats’ body weight). Determining the effect of a selected dose of genistein may enable its application in human cancer prevention or in the improvement of pharmacological treatment.

**Figure 1:**
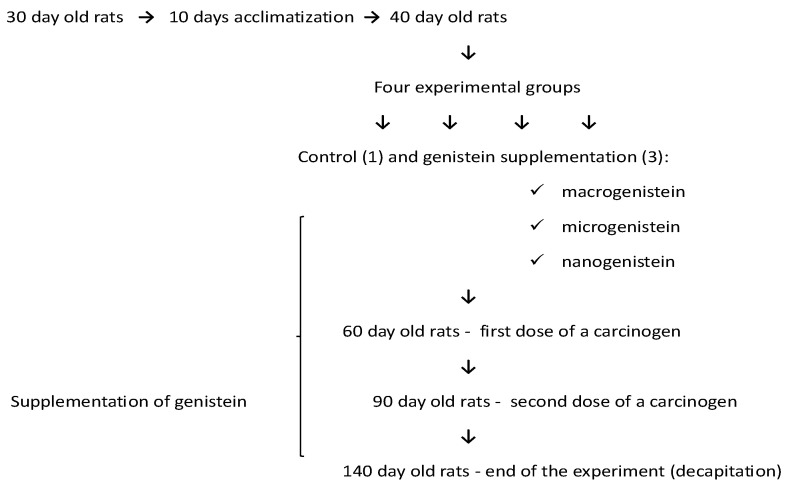
Scheme of experiment design.

The rats were treated twice with DMBA (7,12-dimethyl-1,2-benz(a)anthracene; Sigma-Aldrich, St. Louis, MO, USA) in rapeseed oil (by gavage) to induce mammary cancer (adenocarcinoma). The first treatment was administered at 60 days of age (80 mg/kg body weight), followed by a dose of 40 mg/kg body weight at 90 days of age (Figure 1). Interactive factors were eliminated by applying the same experimental procedure to all rats, i.e., age, experimental time, feed, housing conditions, tumour induction method, and supplementation method. The control group (standard diet—without supplementation) was administered 0.4 mL of water instead of genistein to induce a similar level of stress to the animals in the control group. The animals were examined by palpation during the study to characterize the time course of tumour development. Data on tumour induction in the groups treated with 7,12-dimethyl-1,2-benz(a)anthracene, i.e., the number of tumours and how quickly they appeared, depending on genistein supplementation, were presented in a previous paper [62].

### 2.2. Histopathological and Micromorphometric Examination of Bones

The material for the histopathological examination was right femurs of rats at the age of 20 weeks. Bones were fixed in 10% formalin, decalcified in Shandon TBT-1 for 24 h, and embedded in paraffin. Paraffin sections were stained with haematoxylin and eosin (H&E) [63,64].

Histological evaluation of the specimens was performed using the Olympus BX41 research microscope. Micromorphometric measurements were conducted using Olympus EP 50 software (EPview for Windows OS (64 bit)) for archiving and morphometry of histological images. Measurements were conducted in the diaphysis, including its diameter (width), the thickness of the compact layer, and the total thickness of the diaphysis wall. For each of these features, 50 measurements were taken along the entire length of the femoral bone shaft. Micrometric measurements were provided in micrometres (µm).

### 2.3. Determination of Levels of Elements

#### 2.3.1. Reagents

All solvents and reagents were of the highest commercially available purity. Ultrapure water (resistance 18 MΩ cm^−1^), used to prepare all standards and solutions of samples, was obtained from the Barnstead NANOpure Diamond UV system. Samples were dissolved using 65% HNO3 and 37% HCl, Suprapur (Merck, Darmstadt, Germany). Multi-element solutions of Ag, As, Al, B, Ba, Ca, Cr, Cu, Fe, Mg, Mn, Ni, Rb, Se, Sr, V, and Zn, each at a concentration of 10 mg/L, were purchased from Inorganic Ventures (Christiansburg, VA, USA). Standard stock solutions of Ca, Fe, K, Mg, Na, and Zn at a concentration of 1000 mg/L were purchased from Merck (Germany). The purity of the plasma gas (argon) and collision cell gas (helium) was above 99.999%.

#### 2.3.2. Sampling

The material for analysis was the femurs of the rats. Following resection, the bones were cleaned of soft tissues, i.e., the joint capsule and muscles, and then frozen at −80 °C (as well as other tested tissues). Directly before analysis, the samples were thawed and dried for 10 h at 120 °C, then subjected to mineralization. Each sample of rat bone was placed directly in a hermetically sealed vessel, and 1 mL of HCL and 4 mL of HNO_3_ were added. Samples were digested in a high-pressure laboratory microwave. The heating program was carried out in two steps. In the first step, the temperature was increased linearly from 25 °C to 210 °C over 15 min, and, in the second step, the temperature was maintained at 210 °C for 8 min. Following digestion, the samples were diluted with water to a final volume of 100 mL.

#### 2.3.3. Instrumental Analysis

A 7800 quadrupole ICP-MS (Agilent Technologies, Minato City, Tokyo, Japan), equipped with an octopole collision cell, was used for all trace elements analysed. Measurements were conducted using nickel sampler cones and a skimmer.

Measurements of Ca, Fe, K, Mg, Na, and Zn—at high concentrations of elements—were conducted using flame atomic absorption spectrometers—Solar GF Zeeman and iCE3500 (Thermo Fisher Scientific 168 Third Avenue Waltham, MA, USA)—equipped with lamps with a single hollow cathode, using an air/acetylene flame for the determination of Fe, K, Mg, Na, and Zn and a nitrous oxide/acetylene flame for Ca. The wavelengths for monitoring Ca, Fe, K, Mg, Na, and Zn were 422.7, 248.3, 766.5, 285.2, 589.0, and 213.9 nm, respectively.

Before multielement analysis using ICP-MS and atomic absorption spectrometers, the analytical methods were checked using certificate material (water matrix reference material: EnviroMAT wastewater, high (EU-H), catalogue number 140-025-138, and lot number S160225019 from SCP Science, Quebec, Canada).

### 2.4. Statistical Analysis

Kruskal–Wallis test (followed by post-hoc Dunn test) was used for comparisons of quantitative variables in four groups. Spearman’s correlation coefficient was used to assess correlation between two quantitative variables. Significance level was set to 0.05. All the analyses were conducted in R software, version 4.3.1.

## 3. Results

### 3.1. Analysis of the Weight of Femurs of Rats Fed Different Diets (Standard and Supplemented with Macrogenistein, Microgenistein, or Nanogenistein)

The results of calculation of the ratio of femur weight (g) to body weight (g), in relation to the supplementation used, are presented in Table 1.

### 3.2. Results of Histopathological and Micromorphometric Examination of Rat Femurs

**Figure 2:**
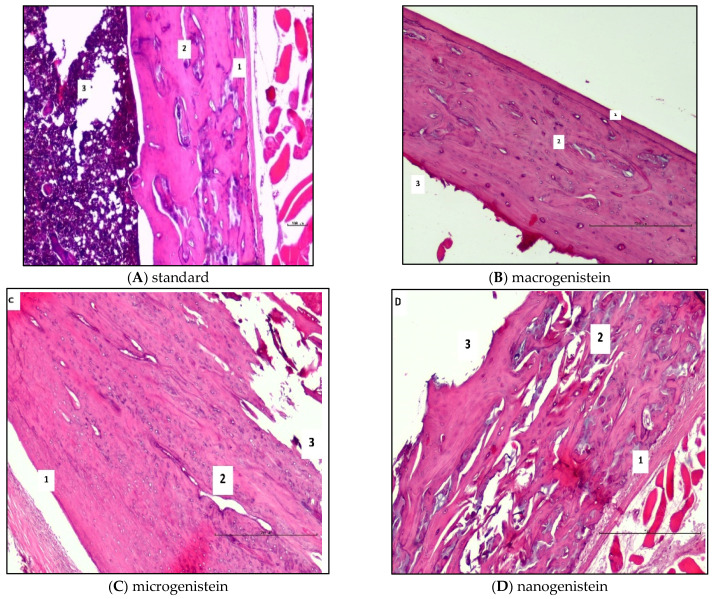
Results of the histopathological analysis of rat femurs in conditions of neoplasia in relation to supplementation (macro-, micro-, or nanogenistein) (1—compact layer; 2—plexiform bone; 3—medullary cavity) (haematoxylin & eosin staining, 20× magnification).

During the collection of biological material for analysis (following sacrifice of the rats), the femurs of the animals supplemented with nanogenistein were observed to be more fragile (breaking in the researcher’s hands under slight pressure). Therefore, histopathological and micromorphometric evaluation of the femurs in relation to the supplementation used was performed (Table 2 and Figure 2). The results of the histological and micromorphometric examination of the bone shaft showed that supplementation with micro- and nanogenistein resulted in significant bone structure disturbances. The width of the bone layer in the animals supplemented with nano- and microgenistein was significantly greater than in those receiving the standard diet. In the bones of rats supplemented with nanogenistein, structural abnormalities such as trauma, cracks, gaps, and porosity were observed. Signs of calcification were detected, as well as the formation of ‘bone islands’. Loose osteoblasts were visible in the histopathological images. In the animals supplemented with nanogenistein, disintegration of the bone tissue was observed.

### 3.3. Analysis of the Content of Minerals in the Femoral Diaphysis of Rats Receiving Various Forms of Genistein: Macrogenistein, Microgenistein, Nanogenistein, or No Supplementation (Standard Diet)

**Figure 3:**
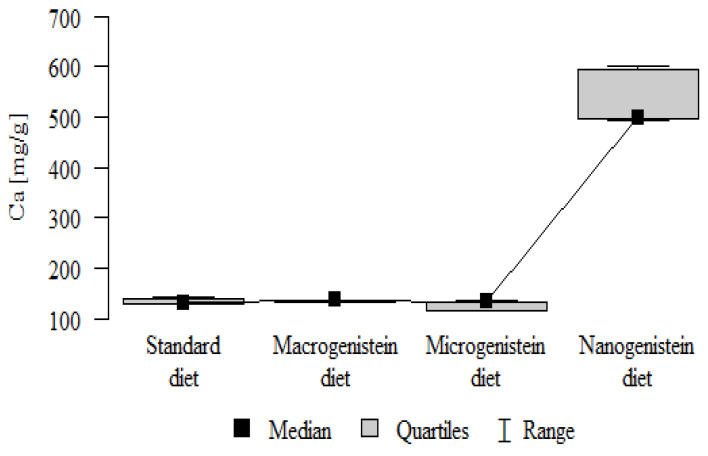
Calcium in the femurs of rats supplemented with various forms of genistein: macrogenistein; microgenistein; nanogenistein; no supplementation (standard diet).

**Figure 4:**
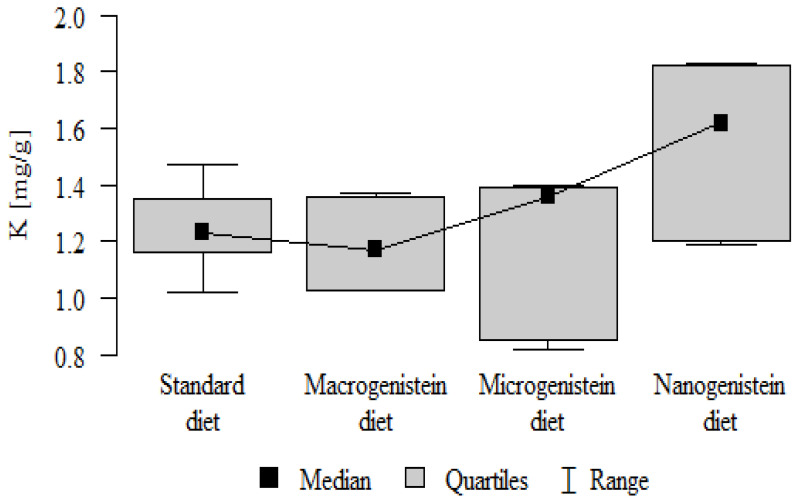
Potassium in the femurs of rats supplemented with various forms of genistein: macrogenistein; microgenistein; nanogenistein; no supplementation (standard diet).

**Figure 5:**
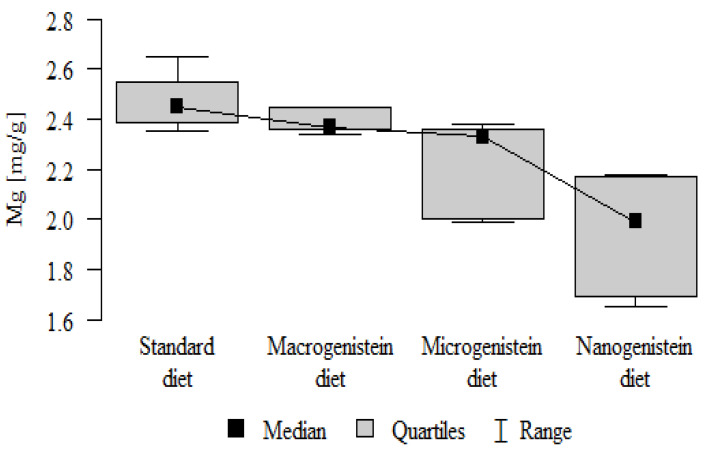
Magnesium in the femurs of rats supplemented with various forms of genistein: macrogenistein; microgenistein; nanogenistein; no supplementation (standard diet).

**Figure 6:**
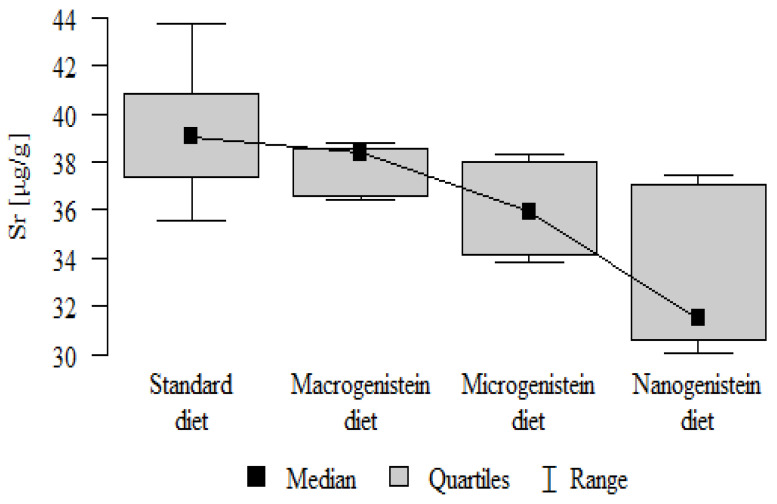
Strontium in the femurs of rats supplemented with various forms of genistein: macrogenistein; microgenistein; nanogenistein; no supplementation (standard diet).

**Figure 7:**
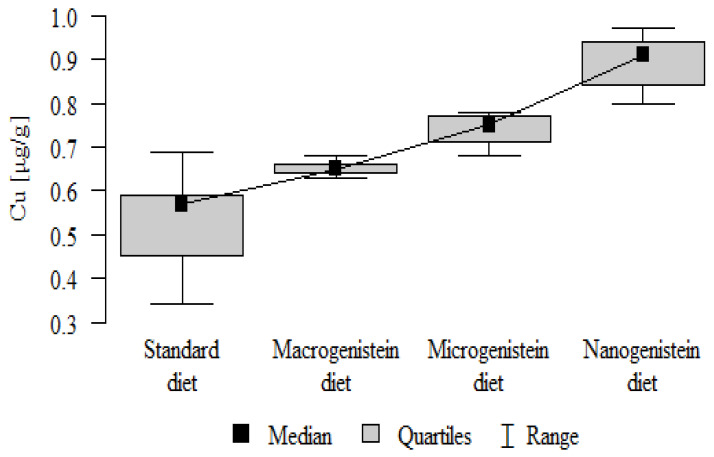
Copper in the femurs of rats supplemented with various forms of genistein: macrogenistein; microgenistein; nanogenistein; no supplementation (standard diet).

**Figure 8:**
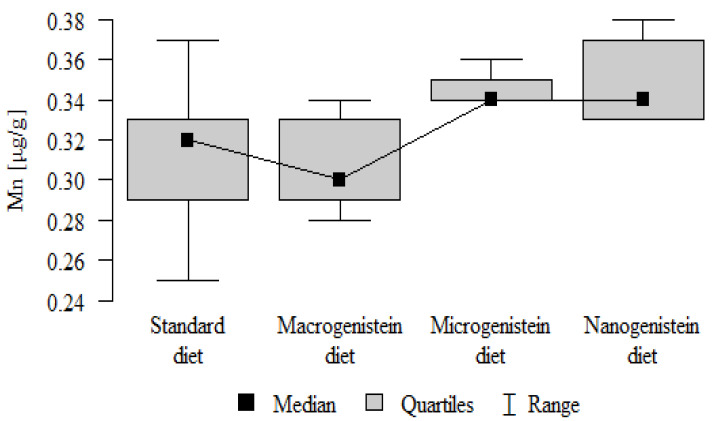
Manganese in the femurs of rats supplemented with various forms of genistein: macrogenistein; microgenistein; nanogenistein; no supplementation (standard diet).

**Figure 9:**
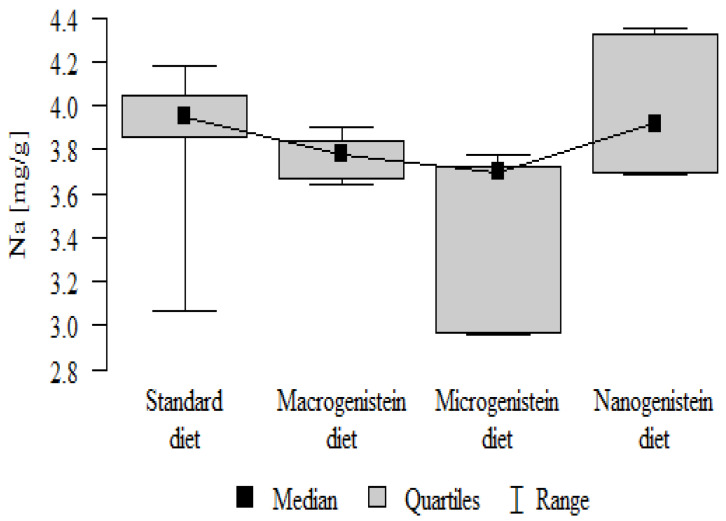
Sodium in the femurs of rats supplemented with various forms of genistein: macrogenistein; microgenistein; nanogenistein; no supplementation (standard diet).

**Figure 10:**
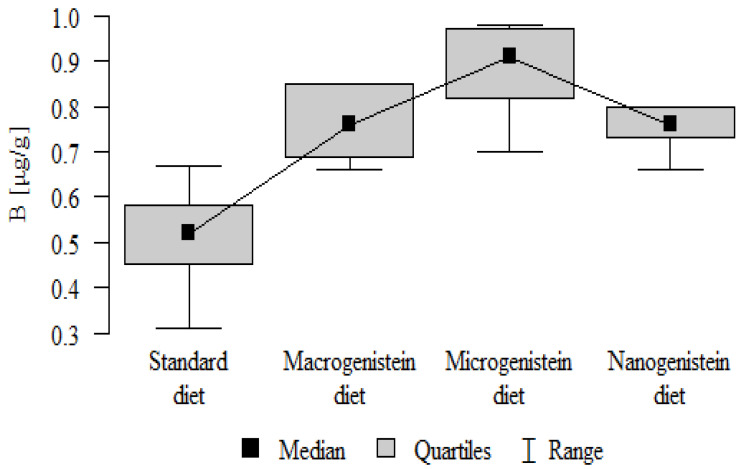
Boron in the femurs of rats supplemented with various forms of genistein: macrogenistein; microgenistein; nanogenistein; no supplementation (standard diet).

**Figure 11:**
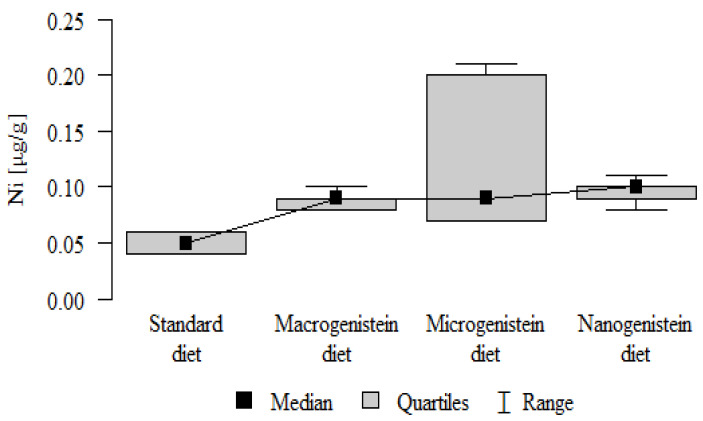
Nickel in the femurs of rats supplemented with various forms of genistein: macrogenistein; microgenistein; nanogenistein; no supplementation (standard diet).

**Figure 12:**
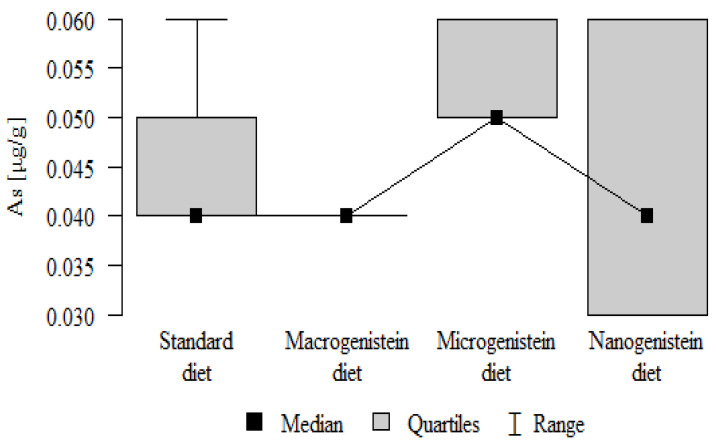
Arsenic in the femurs of rats supplemented with various forms of genistein: macrogenistein; microgenistein; nanogenistein; no supplementation (standard diet).

**Figure 13:**
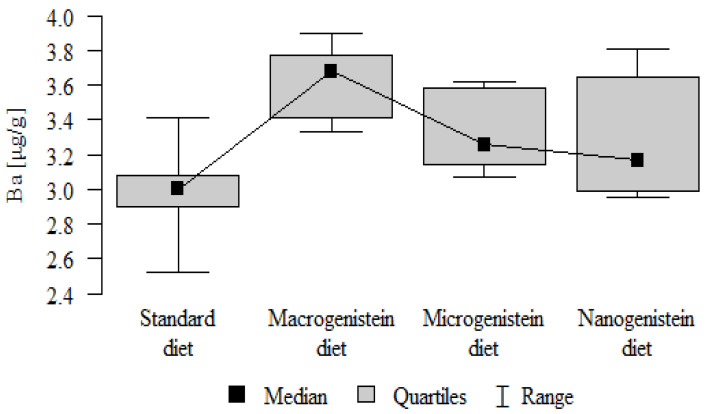
Barium in the femurs of rats supplemented with various forms of genistein: macrogenistein; microgenistein; nanogenistein; no supplementation (standard diet).

The following results were obtained from the analysis:

− Content of Ca and K in the bone tissue was statistically significantly higher in the rats supplemented with nanogenistein than in the other groups (Table 3; Figure 3 and Figure 4).
− Content of Mg and Sr in the bone tissue was significantly higher in the rats without supplementation (standard diet) than in the groups receiving micro- and nanogenistein. The levels of these elements were significantly higher in the bones of rats supplemented with macrogenistein compared to the group supplemented with nanogenistein (Table 3; Figure 5 and Figure 6).
− Content of Cu and Mn was significantly higher in the bone tissue of rats supplemented with nanogenistein and microgenistein compared to the remaining groups (Table 3; Figure 7 and Figure 8).
− Content of Na in the bone tissue was significantly higher in rats on the standard diet and the diet supplemented with nanogenistein than in the bones of rats receiving microgenistein (Table 3; Figure 9).
− Content of B and Ni in the bone tissue was significantly higher in rats supplemented with various forms of genistein than in those receiving the standard diet (Table 3; Figure 10 and Figure 11).
− Content of As in the bones was significantly higher in the group supplemented with microgenistein than in the remaining groups (Table 3; Figure 12).
− Content of Ba in the bones was significantly higher in the group supplemented with macrogenistein than in the groups receiving micro- and nanogenistein and the standard diet. The Ba level was significantly higher in the microgenistein group than in the group on the standard diet (Table 3; Figure 13).
− There were no statistically significant differences in the content of Zn, Fe, Se, V, Cr, and Rb in the bones of the rats (Table 3).

**Table 3:** Mineral composition of rat femurs.

	Diet/Supplementation	Mean	SD	Median	Min	Max	Q1	Q3	*p*
Ca [mg/g]	Standard diet	134.41	5.94	131.78	128.24	143.07	130.07	140.31	*p* < 0.001 *
	Macrogenistein	134.76	2.71	136.03	131.06	137.36	131.40	136.68	nano > macro, micro, stand
	Microgenistein	127.35	8.54	132.60	115.73	134.09	116.18	133.25	
	Nanogenistein	530.73	50.92	499.66	492.92	601.43	495.81	595.52	
**Zn** [mg/g]	Standard diet	0.15	0.01	0.15	0.13	0.16	0.14	0.15	*p* = 0.297
	Macrogenistein	0.14	0.01	0.14	0.13	0.15	0.13	0.14	
	Microgenistein	0.14	0.01	0.13	0.12	0.16	0.12	0.16	
	Nanogenistein	0.16	0.05	0.14	0.13	0.24	0.13	0.22	
K [mg/g]	Standard diet	1.24	0.15	1.23	1.02	1.47	1.16	1.35	*p* = 0.032 *
	Macrogenistein	1.19	0.15	1.17	1.03	1.37	1.03	1.36	nano > micro, stand, macro
	Microgenistein	1.20	0.27	1.36	0.82	1.40	0.85	1.39	
	Nanogenistein	1.55	0.28	1.62	1.19	1.83	1.20	1.82	
Mg [mg/g]	Standard diet	2.47	0.11	2.45	2.35	2.65	2.39	2.55	*p* < 0.001 *
	Macrogenistein	2.39	0.05	2.37	2.34	2.45	2.36	2.45	stand > micro, nano macro > nano
	Microgenistein	2.23	0.18	2.33	1.99	2.38	2.00	2.36	
	Nanogenistein	1.94	0.22	1.99	1.65	2.18	1.69	2.17	
Na [mg/g]	Standard diet	3.87	0.35	3.95	3.07	4.18	3.86	4.05	*p* = 0.005 *
	Macrogenistein	3.77	0.09	3.78	3.64	3.90	3.67	3.84	stand, nano > micro
	Microgenistein	3.47	0.38	3.70	2.96	3.78	2.97	3.72	
	Nanogenistein	3.99	0.28	3.92	3.69	4.35	3.70	4.33	
**Fe** [μg/g]	Standard diet	66.50	12.93	63.79	49.10	94.99	62.30	67.82	*p* = 0.384
	Macrogenistein	61.53	12.87	62.53	45.92	76.24	46.55	75.61	
	Microgenistein	60.87	2.71	59.36	58.70	65.02	58.77	63.21	
	Nanogenistein	55.59	10.89	51.98	44.53	69.78	46.63	69.45	
Cu [μg/g]	Standard diet	0.53	0.11	0.57	0.34	0.69	0.45	0.59	*p* < 0.001 *
	Macrogenistein	0.65	0.02	0.65	0.63	0.68	0.64	0.66	nano, micro > macro, stand
	Microgenistein	0.74	0.04	0.75	0.68	0.78	0.71	0.77	
	Nanogenistein	0.89	0.06	0.91	0.80	0.97	0.84	0.94	
B [μg/g]	Standard diet	0.51	0.12	0.52	0.31	0.67	0.45	0.58	*p* < 0.001 *
	Macrogenistein	0.76	0.08	0.76	0.66	0.85	0.69	0.85	micro, nano, macro > stand
	Microgenistein	0.88	0.10	0.91	0.70	0.98	0.82	0.97	
	Nanogenistein	0.76	0.05	0.76	0.66	0.80	0.73	0.80	
**V** [μg/g]	Standard diet	0.02	0.01	0.03	0.00	0.03	0.02	0.03	*p* = 0.244
	Macrogenistein	0.02	0.00	0.02	0.02	0.03	0.02	0.03	
	Microgenistein	0.02	0.00	0.02	0.02	0.02	0.02	0.02	
	Nanogenistein	0.02	0.00	0.02	0.02	0.03	0.02	0.03	
**Cr** [μg/g]	Standard diet	0.12	0.04	0.12	0.06	0.18	0.11	0.14	*p* = 0.136
	Macrogenistein	0.08	0.04	0.06	0.05	0.13	0.05	0.13	
	Microgenistein	0.10	0.03	0.09	0.07	0.14	0.07	0.14	
	Nanogenistein	0.11	0.00	0.11	0.10	0.11	0.10	0.11	
Mn [μg/g]	Standard diet	0.31	0.04	0.32	0.25	0.37	0.29	0.33	*p* = 0.001 *
	Macrogenistein	0.31	0.02	0.30	0.28	0.34	0.29	0.33	nano, micro > stand, macro
	Microgenistein	0.34	0.01	0.34	0.34	0.36	0.34	0.35	
	Nanogenistein	0.35	0.02	0.34	0.33	0.38	0.33	0.37	
Ni [μg/g]	Standard diet	0.05	0.01	0.05	0.04	0.06	0.04	0.06	*p* < 0.001 *
	Macrogenistein	0.09	0.01	0.09	0.08	0.10	0.08	0.09	nano, micro, macro > stand
	Microgenistein	0.12	0.06	0.09	0.07	0.21	0.07	0.20	
	Nanogenistein	0.10	0.01	0.10	0.08	0.11	0.09	0.10	
As [μg/g]	Standard diet	0.05	0.01	0.04	0.04	0.06	0.04	0.05	*p* = 0.01 *
	Macrogenistein	0.04	0.00	0.04	0.04	0.04	0.04	0.04	micro > macro, stand, nano
	Microgenistein	0.06	0.00	0.05	0.05	0.06	0.05	0.06	
	Nanogenistein	0.04	0.01	0.04	0.03	0.06	0.03	0.06	
**Se** [μg/g]	Standard diet	0.11	0.02	0.11	0.08	0.12	0.10	0.12	*p* = 0.494
	Macrogenistein	0.11	0.00	0.11	0.11	0.12	0.11	0.12	
	Microgenistein	0.11	0.01	0.11	0.09	0.12	0.10	0.12	
	Nanogenistein	0.10	0.02	0.10	0.08	0.12	0.09	0.11	
**Rb** [μg/g]	Standard diet	1.49	0.16	1.48	1.25	1.71	1.41	1.57	*p* = 0.349
	Macrogenistein	1.48	0.20	1.48	1.24	1.74	1.30	1.69	
	Microgenistein	1.51	0.28	1.68	1.13	1.72	1.14	1.71	
	Nanogenistein	1.68	0.35	1.89	1.21	1.95	1.23	1.93	
Sr [μg/g]	Standard diet	39.40	2.93	39.03	35.59	43.77	37.35	40.86	*p* = 0.001 *
	Macrogenistein	37.85	1.00	38.36	36.41	38.79	36.60	38.55	stand > micro, nano macro > nano
	Microgenistein	36.05	1.79	35.94	33.84	38.34	34.15	38.01	
	Nanogenistein	33.03	3.26	31.49	30.05	37.42	30.63	37.08	
Ba [μg/g]	Standard diet	2.98	0.25	3.00	2.52	3.41	2.90	3.08	*p* = 0.001 *
	Macrogenistein	3.63	0.20	3.68	3.33	3.90	3.41	3.77	macro > micro, nano, stand micro > stand
	Microgenistein	3.32	0.22	3.26	3.07	3.62	3.14	3.58	
	Nanogenistein	3.29	0.34	3.17	2.95	3.81	2.99	3.65	

The results of the study were compared using Kruskal–Wallis test (followed by post-hoc Dunn test). The significance level (*) was set to 0.05.

**Figure 14:**
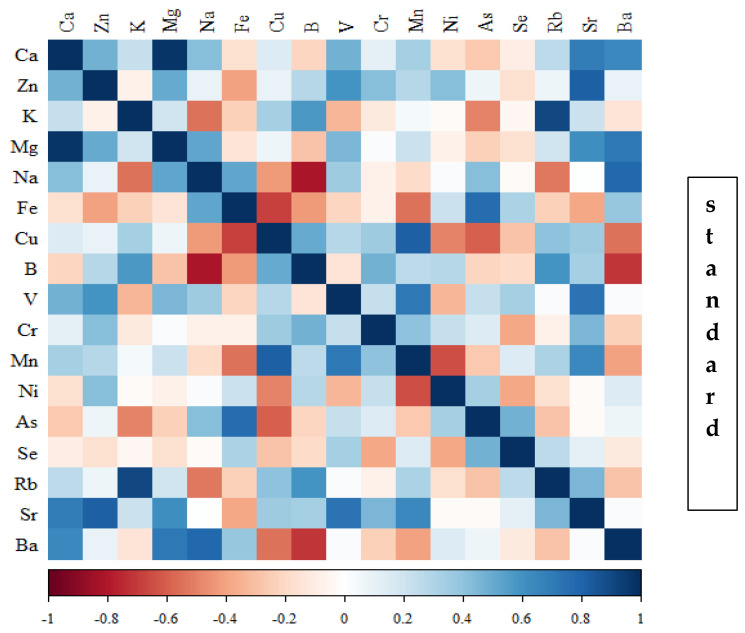
Heat map of correlations of 17 elements in the bones of rats receiving the standard diet (blue indicates positive correlations and red indicates negative correlations).

**Figure 15:**
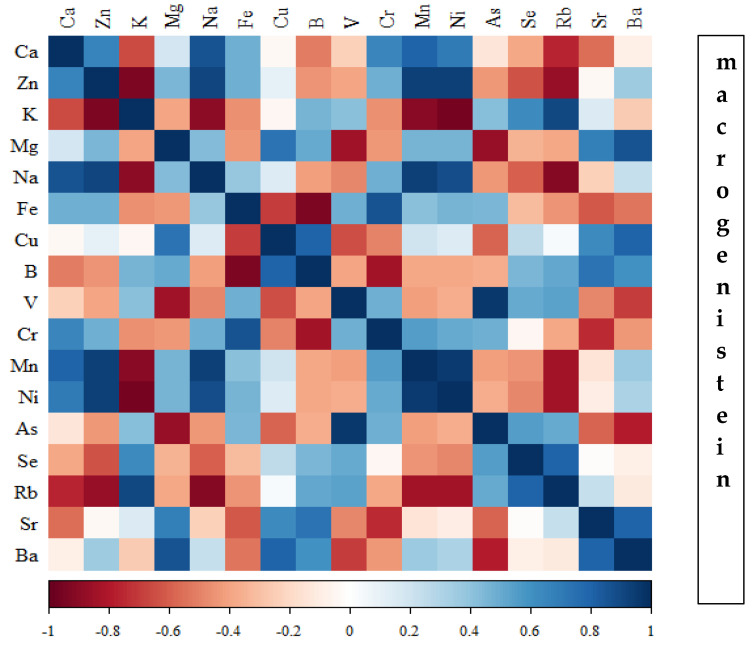
Heat map of correlations of 17 elements in the bones of rats receiving macrogenistein (blue indicates positive correlations and red indicates negative correlations).

**Figure 16:**
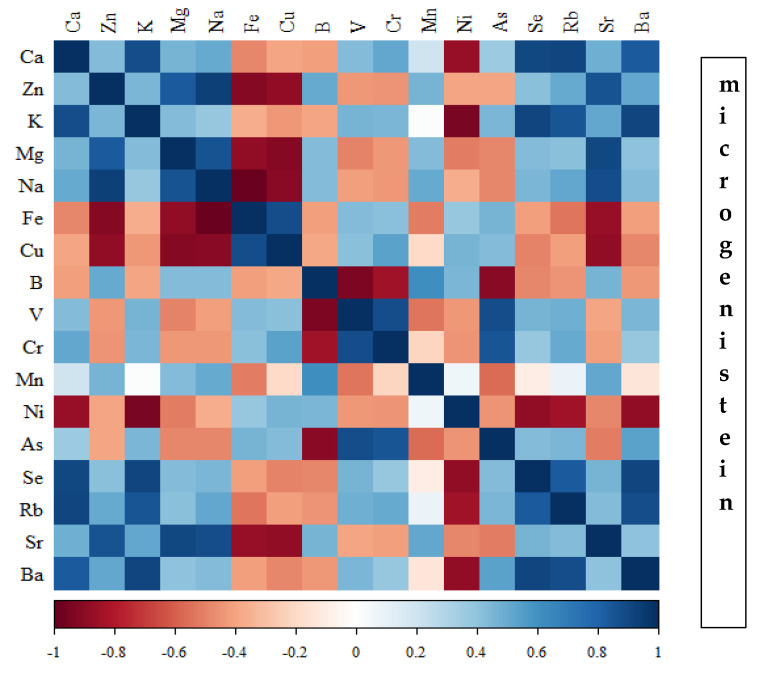
Heat map of correlations of 17 elements in the bones of rats receiving microgenistein (blue indicates positive correlations and red indicates negative correlations).

**Figure 17:**
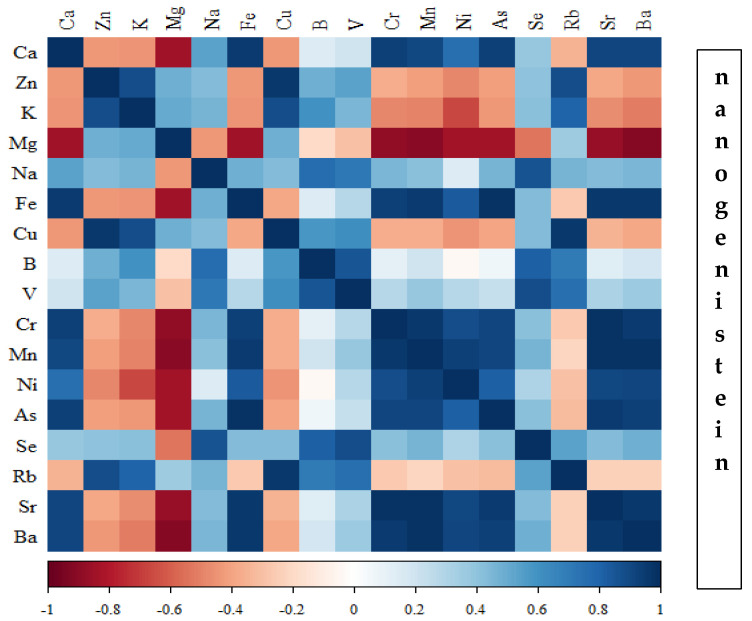
Heat map of correlations of 17 elements in the bones of rats receiving nanogenistein (blue indicates positive correlations and red indicates negative correlations).

Correlations between the content of minerals in the bone tissue of rats depending on the diet used are presented in Figure 14, Figure 15, Figure 16 and Figure 17 and Tables S1–S4 (Supplementary Materials).

In the group of rats receiving the standard diet, there were only a few statistically significant correlations among the elements analysed (Figure 14; Table S1). The majority of the correlations were noted in the group of rats supplemented with nanogenistein (Figure 14, Figure 15, Figure 16 and Figure 17; Tables S1–S4), mainly for Ca, Ni, Fe, Rb, Cr, Ba, As, Sr, and Ni. These elements were negatively correlated with Mg.

## 4. Discussion

The main objective of this research was to expand knowledge of the effects of nanogenistein on the bone tissue of rats in conditions of an induced neoplastic process. Three main conclusions can be drawn from the research. First, bone tissue disintegration took place in the animals supplemented with nanogenistein. Second, we showed a strong negative in vivo effect of administration of nanogenistein on the amount of calcium in the bone tissue, indicating its increased distribution to the bones and changes in micromorphometric parameters. Third, we showed that supplementation of rats with genistein in macro-, micro-, and nanoforms in the conditions of cancer development affects the mineral composition of the bones, resulting not only in their calcification but also in changes in the content of many other elements. It is particularly worth noting the changes in the levels of macroelements in the bone resulting from intake of nanogenistein, especially the most important minerals for this tissue. Thus, there was an enormous increase in calcium by about 300% and a 25% increase in potassium compared to the other groups. The level of magnesium also decreased by about 22%. In the case of microelements, there was an increase in copper (67%), boron (48%), manganese (13%), and nickel (100%) and a 16% decrease in strontium relative to the content of these elements in the bones of rats without supplementation. Interestingly, while supplementation of the diet of rats with microgenistein also caused multiple changes in the mineral composition of the bones, in the case of macroelements, a difference was noted for sodium and magnesium (11% decrease). In the case of microelements, a similar direction of changes was noted as for nanogenistein, i.e., an increase of 39% for copper, 73% for boron, 10% for manganese, 140% for nickel, 20% for arsenic, and 11% for barium, as well as an 8.5% decrease in strontium compared to the group receiving an unsupplemented standard diet. Supplementation of the diet of rats with the classical form of genistein, i.e., macrogenistein, at the dose applied caused differences only in the content of a few microelements The content of boron, nickel, and barium increased by 49%, 80%, and 22%, respectively, relative to the standard group.

Calcium and phosphate ions are crucial factors in maintaining the balance between bone formation and loss. When the calcium balance is disturbed, changes in bone composition appear, leading to bone dysfunction. In our study, this led to increased bone fragility. Therefore, we can conclude that nanogenistein has an unfavourable effect on calcium metabolism via an unknown indirect or direct mechanism. It is possible that the presence of neoplasia exacerbated this effect. Our study took place in conditions of induced mammary cancer. The rate of tumour induction by DMBA was 100%. Histopathological examination confirmed that these were grade 2 adenomas for the standard group and the group receiving genistein in the classical form, and grade 3 in the case of micro- and nanogenistein [62]. In animals supplemented with nanogenistein, the first tumours appeared a few weeks earlier than in the other groups, and, at the end of the experiment, they were larger than in the groups receiving the standard diet or classical genistein. The use of nanotechnology in our study may therefore have translated to a poorer prognosis.

Many reasons for the situation observed in our study may be considered. One may be the fact that the nanoform of genistein was absorbed to such an extent that it reached a level comparable to or higher than that of oestradiol and completely dysregulated calcium homeostasis in the body. The physiological cellular response to oestrogen begins in the cytoplasm, where oestrogen binds to oestrogen receptor alpha or beta. The activated oestrogen–receptor complex then enters the cell nucleus, inducing DNA transcription, binding to specific nucleotide sequences (ERE) in order to induce a physiological response. The oestrogen hormone levels in the body are regulated by negative oestrogen feedback on the hypothalamus and pituitary gland [65]. Clinical examination has shown that even small doses of oestrogen applied for a short time can significantly modulate and impair the resorptive activity of PTH, thereby increasing bone formation and calcium sequestration [66]. According to the authors, oestrogen exerts parallel effects in removing calcium from the blood and sequestering calcium in the bone mineralization process.

It is possible that in our study nanogenistein took over this role or together with oestrogens induced an enhanced response. Alternatively, it may have led to the accumulation of calcium in the bones in other non-oestrogenic mechanisms. The action of sex steroids, which proceeds according to a rapid nongenomic mechanism, involves the activity of several main signalling pathways in the cell, responsible for the integration and transmission of extracellular signals. These include the above-mentioned cascades of MAPK (mitogen-activated protein kinases), tyrosine kinases, and lipid kinases. They are usually associated with the reception of signalling from various growth factors, and their activation in the cell regulates processes such as gene expression, proliferation, and survival [67]. They may also be modified by genistein [19,21,23].

Apart from the classical effects of sex hormones mediated by specific receptors, signals from sex hormones can be induced indirectly. Such a mechanism has been described for 17β-oestradiol (and androgens), transported in the blood together with molecules of sex hormone-binding globulin (SHBG). SHBG is a plasma glycoprotein with strong affinity for sex hormones, regulating their bioavailability and uptake by target cells. Its production in the liver is regulated by sex hormones (oestradiol stimulates it and testosterone inhibits it) [67,68]. Y Musawi et al. [69] discovered that genistein significantly increases SHBG production by Hep-G2 cells.

Finally, genistein may act through the mechanism of influencing intestinal calcium absorption. Vitamin D and 17β-oestradiol are known to increase intestinal absorption of calcium. Research is needed to determine whether isoflavones affect intestinal absorption and reabsorption of calcium by the kidneys. However, active calcium absorption by the duodenum consists of three main stages: calcium influx into the enterocyte, its passage, and excretion from the enterocyte. These depend mainly on calcium transporter 1 (CaT1), calbindin-D9K, and calcium ATPase (PMCA _1b_) in the cell membrane [70]. Oestrogens or hormonal changes (e.g., during pregnancy or lactation) have a pronounced vitamin D-dependent effect at the genome level on the activity of calcium absorption mechanisms in the duodenum, mainly through strong upregulation of the calcium entry channel CaT1. The effect of oestrogen seems to be mediated by Erα alone [70]. Thus, administration of the classical form of genistein and the concentrations attained in the body are not likely to be effective, but, in the case of nanogenistein, its effect on intestinal absorption and reabsorption in the kidneys probably cannot be ruled out.

Oestrogens are generally known to play an important role not only in preserving bone health (a decrease in oestrogen leads to a marked increase in osteoclast activity, which significantly increases bone resorption) [71], but also in calcium homeostasis [57,72,73,74]. During maturation, oestrogen helps in the development of long bones and fusion of epiphyseal growth plates [75]. It protects the bones by inactivating osteoclast activity, preventing osteoporosis in women with oestrogen deficiency as well as in post-menopausal women [76]. Oestrogen thus plays the role of integrator of the calcium system in order to ensure sufficient calcium accumulation in the skeleton during growth and pregnancy, enabling the supply of calcium during lactation and preserving skeletal mechanics [70]. A prolonged reduction in the oestrogen level during menopause leads to the development of osteoporosis [77].

Genistein can have an oestrogenic effect by interacting with oestrogen receptors, especially ER-β (anabolically through stimulation of cell proliferation, induction of expression of the Erα gene in the osteoblasts, and their differentiation and maturation) [78], but it also has ligand-independent effects, including effects on growth factors (e.g., IGF), regulation of signalling on the cell surface by enzymes, such as tyrosine kinase (with a suppressive effect on osteoclasts), and modulation of the activity of endogenous oestrogens [79].

Apart from the oestrogenic effect of isoflavones on bone tissue, genistein aglycone increases calcium retention in the bones (an increase in markers of bone turnover, e.g., bone alkaline phosphatase (BAP) [80]. The literature contains studies in which both resveratrol and genistein exert a beneficial effect on the calcium level in the bone tissue [81]. Resveratrol (at 0.7 mg/kg bw) has been shown to inhibit the decrease in calcium content in the femurs of female rats subjected to ovariectomy [81]. Yamaguchi et al. [82] showed that genistein administered orally at 100 and 300 µg/kg bw for three days causes a significant increase in the calcium level in the femoral metaphysis of young female rats and an increase in the activity of alkaline phosphatase and DNA in femoral metaphyseal bone tissues. Alkaline phosphatase is an enzymatic marker of osteoblasts, while DNA content is an index of the growth and number of bone cells. The use of cycloximide, a protein synthesis inhibitor, negated this beneficial effect, which suggests that genistein acts by increasing the synthesis of the protein components of the bone. According to the authors, this took place through binding to oestrogen receptors and enhancement of the effect of the oestrogen receptor in osteoblastic cells. On the other hand, the serum concentrations of calcium and inorganic phosphates remained unchanged, which may indicate that genistein had no significant effect on intestinal absorption of calcium.

Genistein takes part in calcium homeostasis by mobilizing calcium from the bones to the bloodstream in order to maintain an adequate level in the serum and ensure physiological processes dependent on calcium. On the other hand, the affinity of phytoestrogens for β receptors, as well as the modulation of peroxisome proliferator-activated receptors (PPAR), most likely underly the protective effect of isoflavones in maintaining bone density in post-menopausal women [83]. Genistein has the ability to activate osteoprogenitor cells and osteoblasts at low doses, while high doses have an inhibitory effect, and osteoclast formation is inhibited irrespective of the dosage [84]. These compounds can additionally influence bone tissue metabolism by stimulating synthesis of vitamin D in extrarenal cells [85]. It is also worth noting the hormones regulating calcium homeostasis and the effect of genistein. Thus, PTH mobilizes calcium from the bones and also increases reabsorption of calcium in the kidneys when the serum calcium concentration is low [74,75]. The main target organs for PTH/PTHR1 are the kidneys and bones. In the kidneys, PTH and PTHR1 stimulate calcium reabsorption from the renal tubules, stimulate 1.25-dihydroxycholecalcipherol (1.25-(OH)_2_-vit D), and prevent reabsorption of phosphates. In the bones, PTH/PTHR1 mediates bone resorption by osteoclasts and reduces proliferation of osteoblasts, causing the release of calcium and a decrease in bone mass [74]. In one study, an attempt was made to examine the mechanisms determined by PTH and PTHR1 using genistein in rats whose ovaries had been removed [86]. The authors tried to establish whether genistein modulates the effect of PTH in human SaOS-2 osteoblastic cells in an oestrogen-depleted state and showed that this effect can be achieved by applying genistein at 10^−8^ to 10^−6^ m [86].

The significant role of the thyroid (thyroid hormones and calcitonin produced by the parathyroid glands (PTG)) in bone metabolism was mentioned above. Slight decreases in the serum Ca^2+^ concentration and increases in the P concentration stimulate PTG to synthesize and secrete parathormone (PTH). In the bone and kidneys, PTH binds to its receptors in order to maintain adequate levels of Ca^2+^ and phosphates [87,88,89]. Some studies have shown that genistein reduces the serum concentration of PTH and stimulates the expression of PTHR1 and sodium phosphate cotransporter 2a (NaPi 2a) in the kidneys and expression of PTHR1 in the bone [78,90]. Pantelic et al. [78] administered 30 mg kg^−1^ bw of genistein for three weeks to rats that had undergone orchidectomy and observed that genistein protected the trabecular bone by influencing the activity of thyroid follicular cells (a decrease in circulating thyroid hormones, with no effect on calcitonin production from thyroid C cells). Thus, changes in the concentration of thyroid hormones can play an important role in bone metabolism, and the effect of genistein on this organ can modify the response.

## 5. Conclusions

Supplementation of animals with nanogenistein was shown to cause changes in the mineral composition of femurs, mainly resulting in their calcification. The femurs of these animals had higher content of calcium by nearly 300%. Changes in micromorphometric parameters, resulting in increased bone fragility, were also observed. Administration of nanogenistein was shown to have a negative effect on the amount of calcium in the bone tissue, indicating its increased distribution to the bone.

## Figures and Tables

**Table 1:** Body weight of rats and percentage of femur weight in final body weight (%).

Groups	Final Body Weight ± SD (g)	Femur Weight ± SD (g)	Ratio ± SD (%)
Standard	233.3 ± 17.3	0.969 ± 0.110	0.41 ± 0.03
Macrogenistein	214.5 ± 7.1	0.873 ± 0.087	0.41 ± 0.05
Microgenistein	225.9 ± 13.9	0.993 ± 0.057	0.44 ± 0.05
Nanogenistein	221.1 ± 10.4	0.950 ± 0.048	0.43 ± 0.03

No differences were shown in the body weight of the rats, femur weight, or ratio of femur weight to body weight (%) depending on the supplementation used.

**Table 2:** Micromorphometric measurements of the femoral bone shaft (*n* = 50 measurements). Micrometric measurements were provided in micrometres (µm).

Groups →	Standard X ± SD	Macrogenistein X ± SD	Microgenistein X ± SD	Nanogenistein X ± SD
Diameter of the femoral bone shaft (µm)	1221 ± 69	1141 ± 75 * (↓6%)	1413 ± 67 * (↑15%)	1367 ± 55 * (↑12%)
The thickness of the compact layer (µm)	282 ± 16.47	239 ± 42.59 * (↓15%)	360 ± 121 * (↑28%)	356 ± 110 * (↑26%)
The total thickness of the diaphysis wall. (µm)	32.05 ± 8.53	41.19 ± 4.75 * (↑29%)	25.20 ± 5.65 ** (↓21%)	36.68 ± 13.1 * (↑2%)

The results were compared using Kruskal–Wallis test (followed by post-hoc Dunn test). * *p* < 0.001—statistically significant versus standard group; ** *p* < 0.05—statistically significant versus standard group; ↑—increase); ↓—decrease.

## Data Availability

Data are contained within the article.

## Supplementary Materials

The following supporting information can be downloaded at https://www.mdpi.com/article/10.3390/nu16060912/s1, Table S1: Spearman’s correlation coefficient of elements in the bones of rats receiving the standard diet; Table S2: Spearman’s correlation coefficient of elements in the bones of rats receiving macrogenistein; Table S3: Spearman’s correlation coefficient of elements in the bones of rats receiving microgenistein; Table S4: Spearman’s correlation coefficient of elements in the bones of rats receiving nanogenistein.
