# Supplementary material for: Genistein Supplementation and Bone Health in Breast Cancer in Rats
Source: Nutrients. 2024 Mar 21;16(6):912. doi: 10.3390/nu16060912 (PMC10974594; doi:10.3390/nu16060912)
Supplement: Supplementary file 1 [file nutrients-16-00912-s001.zip › nutrients-2904967-supplementary.pdf]

Table S1. Spearman's correlation coefficient of elements in the bones of rats receiving the standard diet.

	Ca	Zn	K	Mg	Na	Fe	Cu	B	V	Cr	Mn	Ni	As	Se	Rb	Sr	Ba
**Ca**	---	r=0.476, p=0.243	r=0.238, p=0.582	r=0.976, p<0.001 *	r=0.429, p=0.299	r=-0.167, p=0.703	r=0.143, p=0.752	r=-0.214, p=0.619	r=0.476, p=0.243	r=0.119, p=0.793	r=0.333, p=0.428	r=-0.167, p=0.703	r=-0.262, p=0.536	r=-0.095, p=0.84	r=0.262, p=0.536	r=0.69, p=0.069	r=0.643, p=0.096
**Zn**	r=0.476, p=0.243	---	r=-0.071, p=0.882	r=0.5, p=0.216	r=0.095, p=0.84	r=-0.405, p=0.327	r=0.095, p=0.84	r=0.286, p=0.501	r=0.595, p=0.132	r=0.429, p=0.299	r=0.286, p=0.501	r=0.429, p=0.299	r=0.071, p=0.882	r=-0.167, p=0.703	r=0.071, p=0.882	r=0.81, p=0.022 *	r=0.095, p=0.84
**K**	r=0.238, p=0.582	r=-0.071, p=0.882	---	r=0.19, p=0.665	r=-0.548, p=0.171	r=-0.238, p=0.582	r=0.333, p=0.428	r=0.571, p=0.151	r=-0.333, p=0.428	r=-0.119, p=0.793	r=0.048, p=0.935	r=-0.024, p=0.977	r=-0.5, p=0.216	r=-0.048, p=0.935	r=0.905, p=0.005 *	r=0.214, p=0.619	r=-0.143, p=0.752
**Mg**	r=0.976, p<0.001 *	r=0.5, p=0.216	r=0.19, p=0.665	---	r=0.524, p=0.197	r=-0.143, p=0.752	r=0.071, p=0.882	r=-0.286, p=0.501	r=0.452, p=0.267	r=0.024, p=0.977	r=0.214, p=0.619	r=-0.071, p=0.882	r=-0.238, p=0.582	r=-0.167, p=0.703	r=0.19, p=0.665	r=0.619, p=0.115	r=0.714, p=0.058
**Na**	r=0.429, p=0.299	r=0.095, p=0.84	r=-0.548, p=0.171	r=0.524, p=0.197	---	r=0.524, p=0.197	r=-0.429, p=0.299	r=-0.81, p=0.022 *	r=0.357, p=0.389	r=-0.071, p=0.882	r=-0.19, p=0.665	r=0.024, p=0.977	r=0.429, p=0.299	r=-0.024, p=0.977	r=-0.524, p=0.197	r=0, p=1	r=0.786, p=0.028 *
**Fe**	r=-0.167, p=0.703	r=-0.405, p=0.327	r=-0.238, p=0.582	r=-0.143, p=0.752	r=0.524, p=0.197	---	r=-0.69, p=0.069	r=-0.429, p=0.299	r=-0.214, p=0.619	r=-0.071, p=0.882	r=-0.548, p=0.171	r=0.214, p=0.619	r=0.762, p=0.037 *	r=0.31, p=0.462	r=-0.238, p=0.582	r=-0.381, p=0.36	r=0.381, p=0.36
**Cu**	r=0.143, p=0.752	r=0.095, p=0.84	r=0.333, p=0.428	r=0.071, p=0.882	r=-0.429, p=0.299	r=-0.69, p=0.069	---	r=0.5, p=0.216	r=0.286, p=0.501	r=0.357, p=0.389	r=0.81, p=0.022 *	r=-0.5, p=0.216	r=-0.595, p=0.132	r=-0.286, p=0.501	r=0.405, p=0.327	r=0.357, p=0.389	r=-0.548, p=0.171
**B**	r=-0.214, p=0.619	r=0.286, p=0.501	r=0.571, p=0.151	r=-0.286, p=0.501	r=-0.81, p=0.022 *	r=-0.429, p=0.299	r=0.5, p=0.216	---	r=-0.143, p=0.752	r=0.476, p=0.243	r=0.262, p=0.536	r=0.286, p=0.501	r=-0.214, p=0.619	r=-0.19, p=0.665	r=0.595, p=0.132	r=0.333, p=0.428	r=-0.714, p=0.058
**V**	r=0.476, p=0.243	r=0.595, p=0.132	r=-0.333, p=0.428	r=0.452, p=0.267	r=0.357, p=0.389	r=-0.214, p=0.619	r=0.286, p=0.501	r=-0.143, p=0.752	---	r=0.238, p=0.582	r=0.714, p=0.058	r=-0.333, p=0.428	r=0.238, p=0.582	r=0.333, p=0.428	r=0.024, p=0.977	r=0.738, p=0.046 *	r=0.024, p=0.977
**Cr**	r=0.119, p=0.793	r=0.429, p=0.299	r=-0.119, p=0.793	r=0.024, p=0.977	r=-0.071, p=0.882	r=-0.071, p=0.882	r=0.357, p=0.389	r=0.476, p=0.243	r=0.238, p=0.582	---	r=0.405, p=0.327	r=0.238, p=0.582	r=0.143, p=0.752	r=-0.381, p=0.36	r=-0.071, p=0.882	r=0.452, p=0.267	r=-0.238, p=0.582
**Mn**	r=0.333, p=0.428	r=0.286, p=0.501	r=0.048, p=0.935	r=0.214, p=0.619	r=-0.19, p=0.665	r=-0.548, p=0.171	r=0.81, p=0.022 *	r=0.262, p=0.536	r=0.714, p=0.058	r=0.405, p=0.327	---	r=-0.643, p=0.096	r=-0.262, p=0.536	r=0.143, p=0.752	r=0.31, p=0.462	r=0.643, p=0.096	r=-0.405, p=0.327
**Ni**	r=-0.167, p=0.703	r=0.429, p=0.299	r=-0.024, p=0.977	r=-0.071, p=0.882	r=0.024, p=0.977	r=0.214, p=0.619	r=-0.5, p=0.216	r=0.286, p=0.501	r=-0.333, p=0.428	r=0.238, p=0.582	r=-0.643, p=0.096	---	r=0.333, p=0.428	r=-0.381, p=0.36	r=-0.167, p=0.703	r=-0.024, p=0.977	r=0.143, p=0.752
**As**	r=-0.262, p=0.536	r=0.071, p=0.882	r=-0.5, p=0.216	r=-0.238, p=0.582	r=0.429, p=0.299	r=0.762, p=0.037 *	r=-0.595, p=0.132	r=-0.214, p=0.619	r=0.238, p=0.582	r=0.143, p=0.752	r=-0.262, p=0.536	r=0.333, p=0.428	---	r=0.476, p=0.243	r=-0.286, p=0.501	r=-0.024, p=0.977	r=0.071, p=0.882
**Se**	r=-0.095, p=0.84	r=-0.167, p=0.703	r=-0.048, p=0.935	r=-0.167, p=0.703	r=-0.024, p=0.977	r=0.31, p=0.462	r=-0.286, p=0.501	r=-0.19, p=0.665	r=0.333, p=0.428	r=-0.381, p=0.36	r=0.143, p=0.752	r=-0.381, p=0.36	r=0.476, p=0.243	---	r=0.262, p=0.536	r=0.119, p=0.793	r=-0.119, p=0.793

	Ca	Zn	K	Mg	Na	Fe	Cu	B	V	Cr	Mn	Ni	As	Se	Rb	Sr	Ba
**Rb**	r=0.262, p=0.536	r=0.071, p=0.882	r=0.905, p=0.005 *	r=0.19, p=0.665	r=-0.524, p=0.197	r=-0.238, p=0.582	r=0.405, p=0.327	r=0.595, p=0.132	r=0.024, p=0.977	r=-0.071, p=0.882	r=0.31, p=0.462	r=-0.167, p=0.703	r=-0.286, p=0.501	r=0.262, p=0.536	---	r=0.452, p=0.267	r=-0.286, p=0.501
**Sr**	r=0.69, p=0.069	r=0.81, p=0.022 *	r=0.214, p=0.619	r=0.619, p=0.115	r=0, p=1	r=-0.381, p=0.36	r=0.357, p=0.389	r=0.333, p=0.428	r=0.738, p=0.046 *	r=0.452, p=0.267	r=0.643, p=0.096	r=-0.024, p=0.977	r=-0.024, p=0.977	r=0.119, p=0.793	r=0.452, p=0.267	---	r=0.024, p=0.977
**Ba**	r=0.643, p=0.096	r=0.095, p=0.84	r=-0.143, p=0.752	r=0.714, p=0.058	r=0.786, p=0.028 *	r=0.381, p=0.36	r=-0.548, p=0.171	r=-0.714, p=0.058	r=0.024, p=0.977	r=-0.238, p=0.582	r=-0.405, p=0.327	r=0.143, p=0.752	r=0.071, p=0.882	r=-0.119, p=0.793	r=-0.286, p=0.501	r=0.024, p=0.977	---

r - Spearman's correlation coefficient
* statistically significant (p<0.05)

Table S2. Spearman's correlation coefficient of elements in the bone of rats receiving macrogenistein.

	Ca	Zn	K	Mg	Na	Fe	Cu	B	V	Cr	Mn	Ni	As	Se	Rb	Sr	Ba
**Ca**	---	r=0.667, p=0.059	r=-0.653, p=0.057	r=0.183, p=0.644	r=0.867, p=0.005 *	r=0.483, p=0.194	r=-0.033, p=0.948	r=-0.517, p=0.162	r=-0.233, p=0.552	r=0.65, p=0.067	r=0.8, p=0.014 *	r=0.7, p=0.043 *	r=-0.133, p=0.744	r=-0.383, p=0.312	r=-0.767, p=0.021 *	r=-0.55, p=0.133	r=-0.083, p=0.843
**Zn**	r=0.667, p=0.059	---	r=-0.937, p<0.001 *	r=0.45, p=0.23	r=0.917, p=0.001 *	r=0.483, p=0.194	r=0.1, p=0.81	r=-0.45, p=0.23	r=-0.4, p=0.291	r=0.483, p=0.194	r=0.933, p=0.001 *	r=0.933, p=0.001 *	r=-0.433, p=0.25	r=-0.633, p=0.076	r=-0.867, p=0.005 *	r=-0.033, p=0.948	r=0.35, p=0.359
**K**	r=-0.653, p=0.057	r=-0.937, p<0.001 *	---	r=-0.393, p=0.295	r=-0.895, p=0.001 *	r=-0.452, p=0.222	r=-0.042, p=0.915	r=0.46, p=0.213	r=0.418, p=0.262	r=-0.452, p=0.222	r=-0.904, p=0.001 *	r=-0.954, p<0.001 *	r=0.427, p=0.252	r=0.636, p=0.066	r=0.904, p=0.001 *	r=0.151, p=0.699	r=-0.251, p=0.515
**Mg**	r=0.183, p=0.644	r=0.45, p=0.23	r=-0.393, p=0.295	---	r=0.433, p=0.25	r=-0.433, p=0.25	r=0.733, p=0.031 *	r=0.5, p=0.178	r=-0.85, p=0.006 *	r=-0.433, p=0.25	r=0.467, p=0.213	r=0.467, p=0.213	r=-0.867, p=0.005 *	r=-0.35, p=0.359	r=-0.383, p=0.312	r=0.683, p=0.05	r=0.867, p=0.005 *
**Na**	r=0.867, p=0.005 *	r=0.917, p=0.001 *	r=-0.895, p=0.001 *	r=0.433, p=0.25	---	r=0.383, p=0.312	r=0.15, p=0.708	r=-0.417, p=0.27	r=-0.483, p=0.194	r=0.483, p=0.194	r=0.933, p=0.001 *	r=0.883, p=0.003 *	r=-0.433, p=0.25	r=-0.6, p=0.097	r=-0.917, p=0.001 *	r=-0.233, p=0.552	r=0.233, p=0.552
**Fe**	r=0.483, p=0.194	r=0.483, p=0.194	r=-0.452, p=0.222	r=-0.433, p=0.25	r=0.383, p=0.312	---	r=-0.7, p=0.043 *	r=-0.933, p=0.001 *	r=0.483, p=0.194	r=0.867, p=0.005 *	r=0.417, p=0.27	r=0.467, p=0.213	r=0.45, p=0.23	r=-0.317, p=0.41	r=-0.45, p=0.23	r=-0.617, p=0.086	r=-0.533, p=0.148
**Cu**	r=-0.033, p=0.948	r=0.1, p=0.81	r=-0.042, p=0.915	r=0.733, p=0.031 *	r=0.15, p=0.708	r=-0.7, p=0.043 *	---	r=0.8, p=0.014 *	r=-0.65, p=0.067	r=-0.5, p=0.178	r=0.2, p=0.613	r=0.15, p=0.708	r=-0.583, p=0.108	r=0.25, p=0.521	r=0.033, p=0.948	r=0.633, p=0.076	r=0.8, p=0.014 *
**B**	r=-0.517, p=0.162	r=-0.45, p=0.23	r=0.46, p=0.213	r=0.5, p=0.178	r=-0.417, p=0.27	r=-0.933, p=0.001 *	r=0.8, p=0.014 *	---	r=-0.4, p=0.291	r=-0.833, p=0.008 *	r=-0.383, p=0.312	r=-0.383, p=0.312	r=-0.367, p=0.336	r=0.45, p=0.23	r=0.517, p=0.162	r=0.733, p=0.031 *	r=0.6, p=0.097
**V**	r=-0.233, p=0.552	r=-0.4, p=0.291	r=0.418, p=0.262	r=-0.85, p=0.006 *	r=-0.483, p=0.194	r=0.483, p=0.194	r=-0.65, p=0.067	r=-0.4, p=0.291	---	r=0.483, p=0.194	r=-0.417, p=0.27	r=-0.367, p=0.336	r=0.967, p<0.001 *	r=0.5, p=0.178	r=0.533, p=0.148	r=-0.483, p=0.194	r=-0.7, p=0.043 *
**Cr**	r=0.65, p=0.067	r=0.483, p=0.194	r=-0.452, p=0.222	r=-0.433, p=0.25	r=0.483, p=0.194	r=0.867, p=0.005 *	r=-0.5, p=0.178	r=-0.833, p=0.008 *	r=0.483, p=0.194	---	r=0.55, p=0.133	r=0.5, p=0.178	r=0.483, p=0.194	r=-0.05, p=0.912	r=-0.383, p=0.312	r=-0.75, p=0.025 *	r=-0.433, p=0.25
**Mn**	r=0.8, p=0.014 *	r=0.933, p=0.001 *	r=-0.904, p=0.001 *	r=0.467, p=0.213	r=0.933, p=0.001 *	r=0.417, p=0.27	r=0.2, p=0.613	r=-0.383, p=0.312	r=-0.417, p=0.27	r=0.55, p=0.133	---	r=0.95, p<0.001 *	r=-0.417, p=0.27	r=-0.45, p=0.23	r=-0.833, p=0.008 *	r=-0.15, p=0.708	r=0.367, p=0.336
**Ni**	r=0.7, p=0.043 *	r=0.933, p=0.001 *	r=-0.954, p<0.001 *	r=0.467, p=0.213	r=0.883, p=0.003 *	r=0.467, p=0.213	r=0.15, p=0.708	r=-0.383, p=0.312	r=-0.367, p=0.336	r=0.5, p=0.178	r=0.95, p<0.001 *	---	r=-0.367, p=0.336	r=-0.483, p=0.194	r=-0.833, p=0.008 *	r=-0.1, p=0.81	r=0.317, p=0.41
**As**	r=-0.133, p=0.744	r=-0.433, p=0.25	r=0.427, p=0.252	r=-0.867, p=0.005 *	r=-0.433, p=0.25	r=0.45, p=0.23	r=-0.583, p=0.108	r=-0.367, p=0.336	r=0.967, p<0.001 *	r=0.483, p=0.194	r=-0.417, p=0.27	r=-0.367, p=0.336	---	r=0.55, p=0.133	r=0.5, p=0.178	r=-0.583, p=0.108	r=-0.783, p=0.017 *
**Se**	r=-0.383, p=0.312	r=-0.633, p=0.076	r=0.636, p=0.066	r=-0.35, p=0.359	r=-0.6, p=0.097	r=-0.317, p=0.41	r=0.25, p=0.521	r=0.45, p=0.23	r=0.5, p=0.178	r=-0.05, p=0.912	r=-0.45, p=0.23	r=-0.483, p=0.194	r=0.55, p=0.133	---	r=0.8, p=0.014 *	r=-0.017, p=0.982	r=-0.083, p=0.843
**Rb**	r=-0.767, p=0.021 *	r=-0.867, p=0.005 *	r=0.904, p=0.001 *	r=-0.383, p=0.312	r=-0.917, p=0.001 *	r=-0.45, p=0.23	r=0.033, p=0.948	r=0.517, p=0.162	r=0.533, p=0.148	r=-0.383, p=0.312	r=-0.833, p=0.008 *	r=-0.833, p=0.008 *	r=0.5, p=0.178	r=0.8, p=0.014 *	---	r=0.233, p=0.552	r=-0.117, p=0.776

	Ca	Zn	K	Mg	Na	Fe	Cu	B	V	Cr	Mn	Ni	As	Se	Rb	Sr	Ba
**Sr**	r=-0.55, p=0.133	r=-0.033, p=0.948	r=0.151, p=0.699	r=0.683, p=0.05	r=-0.233, p=0.552	r=-0.617, p=0.086	r=0.633, p=0.076	r=0.733, p=0.031 *	r=-0.483, p=0.194	r=-0.75, p=0.025 *	r=-0.15, p=0.708	r=-0.1, p=0.81	r=-0.583, p=0.108	r=-0.017, p=0.982	r=0.233, p=0.552	---	r=0.8, p=0.014 *
**Ba**	r=-0.083, p=0.843	r=0.35, p=0.359	r=-0.251, p=0.515	r=0.867, p=0.005 *	r=0.233, p=0.552	r=-0.533, p=0.148	r=0.8, p=0.014 *	r=0.6, p=0.097	r=-0.7, p=0.043 *	r=-0.433, p=0.25	r=0.367, p=0.336	r=0.317, p=0.41	r=-0.783, p=0.017 *	r=-0.083, p=0.843	r=-0.117, p=0.776	r=0.8, p=0.014 *	---

r - Spearman's correlation coefficient

* statistically significant (p<0.05)

Table S3. Spearman's correlation coefficient of elements in the bone of rats receiving microgenistein.

	Ca	Zn	K	Mg	Na	Fe	Cu	B	V	Cr	Mn	Ni	As	Se	Rb	Sr	Ba
**Ca**	---	r=0.433, p=0.25	r=0.883, p=0.003 *	r=0.467, p=0.213	r=0.5, p=0.178	r=-0.483, p=0.194	r=-0.4, p=0.291	r=-0.417, p=0.27	r=0.433, p=0.25	r=0.517, p=0.162	r=0.2, p=0.613	r=-0.867, p=0.005 *	r=0.367, p=0.336	r=0.9, p=0.002 *	r=0.917, p=0.001 *	r=0.483, p=0.194	r=0.833, p=0.008 *
**Zn**	r=0.433, p=0.25	---	r=0.45, p=0.23	r=0.833, p=0.008 *	r=0.933, p=0.001 *	r=-0.917, p=0.001 *	r=-0.883, p=0.003 *	r=0.5, p=0.178	r=-0.433, p=0.25	r=-0.45, p=0.23	r=0.467, p=0.213	r=-0.4, p=0.291	r=-0.4, p=0.291	r=0.417, p=0.27	r=0.5, p=0.178	r=0.867, p=0.005 *	r=0.517, p=0.162
**K**	r=0.883, p=0.003 *	r=0.45, p=0.23	---	r=0.433, p=0.25	r=0.383, p=0.312	r=-0.367, p=0.336	r=-0.433, p=0.25	r=-0.4, p=0.291	r=0.467, p=0.213	r=0.45, p=0.23	r=0.017, p=0.982	r=-0.95, p<0.001 *	r=0.45, p=0.23	r=0.917, p=0.001 *	r=0.85, p=0.006 *	r=0.517, p=0.162	r=0.917, p=0.001 *
**Mg**	r=0.467, p=0.213	r=0.833, p=0.008 *	r=0.433, p=0.25	---	r=0.867, p=0.005 *	r=-0.883, p=0.003 *	r=-0.917, p=0.001 *	r=0.433, p=0.25	r=-0.5, p=0.178	r=-0.433, p=0.25	r=0.433, p=0.25	r=-0.517, p=0.162	r=-0.483, p=0.194	r=0.433, p=0.25	r=0.417, p=0.27	r=0.9, p=0.002 *	r=0.4, p=0.291
**Na**	r=0.5, p=0.178	r=0.933, p=0.001 *	r=0.383, p=0.312	r=0.867, p=0.005 *	---	r=-0.983, p<0.001 *	r=-0.9, p=0.002 *	r=0.433, p=0.25	r=-0.417, p=0.27	r=-0.433, p=0.25	r=0.5, p=0.178	r=-0.367, p=0.336	r=-0.483, p=0.194	r=0.45, p=0.23	r=0.517, p=0.162	r=0.883, p=0.003 *	r=0.433, p=0.25
**Fe**	r=-0.483, p=0.194	r=-0.917, p=0.001 *	r=-0.367, p=0.336	r=-0.883, p=0.003 *	r=-0.983, p<0.001 *	---	r=0.883, p=0.003 *	r=-0.417, p=0.27	r=0.433, p=0.25	r=0.417, p=0.27	r=-0.517, p=0.162	r=0.383, p=0.312	r=0.467, p=0.213	r=-0.417, p=0.27	r=-0.533, p=0.148	r=-0.867, p=0.005 *	r=-0.417, p=0.27
**Cu**	r=-0.4, p=0.291	r=-0.883, p=0.003 *	r=-0.433, p=0.25	r=-0.917, p=0.001 *	r=-0.9, p=0.002 *	r=0.883, p=0.003 *	---	r=-0.383, p=0.312	r=0.417, p=0.27	r=0.533, p=0.148	r=-0.2, p=0.613	r=0.467, p=0.213	r=0.433, p=0.25	r=-0.5, p=0.178	r=-0.417, p=0.27	r=-0.883, p=0.003 *	r=-0.483, p=0.194
**B**	r=-0.417, p=0.27	r=0.5, p=0.178	r=-0.4, p=0.291	r=0.433, p=0.25	r=0.433, p=0.25	r=-0.417, p=0.27	r=-0.383, p=0.312	---	r=-0.933, p=0.001 *	r=-0.85, p=0.006 *	r=0.617, p=0.086	r=0.45, p=0.23	r=-0.9, p=0.002 *	r=-0.483, p=0.194	r=-0.45, p=0.23	r=0.467, p=0.213	r=-0.433, p=0.25
**V**	r=0.433, p=0.25	r=-0.433, p=0.25	r=0.467, p=0.213	r=-0.5, p=0.178	r=-0.417, p=0.27	r=0.433, p=0.25	r=0.417, p=0.27	r=-0.933, p=0.001 *	---	r=0.883, p=0.003 *	r=-0.533, p=0.148	r=-0.433, p=0.25	r=0.883, p=0.003 *	r=0.467, p=0.213	r=0.483, p=0.194	r=-0.4, p=0.291	r=0.45, p=0.23
**Cr**	r=0.517, p=0.162	r=-0.45, p=0.23	r=0.45, p=0.23	r=-0.433, p=0.25	r=-0.433, p=0.25	r=0.417, p=0.27	r=0.533, p=0.148	r=-0.85, p=0.006 *	r=0.883, p=0.003 *	---	r=-0.217, p=0.581	r=-0.45, p=0.23	r=0.85, p=0.006 *	r=0.383, p=0.312	r=0.5, p=0.178	r=-0.417, p=0.27	r=0.383, p=0.312
**Mn**	r=0.2, p=0.613	r=0.467, p=0.213	r=0.017, p=0.982	r=0.433, p=0.25	r=0.5, p=0.178	r=-0.517, p=0.162	r=-0.2, p=0.613	r=0.617, p=0.086	r=-0.533, p=0.148	r=-0.217, p=0.581	---	r=0.067, p=0.88	r=-0.567, p=0.121	r=-0.1, p=0.81	r=0.083, p=0.843	r=0.517, p=0.162	r=-0.133, p=0.744
**Ni**	r=-0.867, p=0.005 *	r=-0.4, p=0.291	r=-0.95, p<0.001 *	r=-0.517, p=0.162	r=-0.367, p=0.336	r=0.383, p=0.312	r=0.467, p=0.213	r=0.45, p=0.23	r=-0.433, p=0.25	r=-0.45, p=0.23	r=0.067, p=0.88	---	r=-0.45, p=0.23	r=-0.883, p=0.003 *	r=-0.85, p=0.006 *	r=-0.483, p=0.194	r=-0.883, p=0.003 *
**As**	r=0.367, p=0.336	r=-0.4, p=0.291	r=0.45, p=0.23	r=-0.483, p=0.194	r=-0.483, p=0.194	r=0.467, p=0.213	r=0.433, p=0.25	r=-0.9, p=0.002 *	r=0.883, p=0.003 *	r=0.85, p=0.006 *	r=-0.567, p=0.121	r=-0.45, p=0.23	---	r=0.433, p=0.25	r=0.45, p=0.23	r=-0.517, p=0.162	r=0.533, p=0.148
**Se**	r=0.9, p=0.002 *	r=0.417, p=0.27	r=0.917, p=0.001 *	r=0.433, p=0.25	r=0.45, p=0.23	r=-0.417, p=0.27	r=-0.5, p=0.178	r=-0.483, p=0.194	r=0.467, p=0.213	r=0.383, p=0.312	r=-0.1, p=0.81	r=-0.883, p=0.003 *	r=0.433, p=0.25	---	r=0.833, p=0.008 *	r=0.467, p=0.213	r=0.917, p=0.001 *

	Ca	Zn	K	Mg	Na	Fe	Cu	B	V	Cr	Mn	Ni	As	Se	Rb	Sr	Ba
**Rb**	r=0.917,<br>p=0.001 *	r=0.5,<br>p=0.178	r=0.85,<br>p=0.006 *	r=0.417,<br>p=0.27	r=0.517,<br>p=0.162	r=-0.533,<br>p=0.148	r=-0.417,<br>p=0.27	r=-0.45,<br>p=0.23	r=0.483,<br>p=0.194	r=0.5,<br>p=0.178	r=0.083,<br>p=0.843	r=-0.85,<br>p=0.006 *	r=0.45,<br>p=0.23	r=0.833,<br>p=0.008 *	---	r=0.433,<br>p=0.25	r=0.883,<br>p=0.003 *
**Sr**	r=0.483,<br>p=0.194	r=0.867,<br>p=0.005 *	r=0.517,<br>p=0.162	r=0.9,<br>p=0.002 *	r=0.883,<br>p=0.003 *	r=-0.867,<br>p=0.005 *	r=-0.883,<br>p=0.003 *	r=0.467,<br>p=0.213	r=-0.4,<br>p=0.291	r=-0.417,<br>p=0.27	r=0.517,<br>p=0.162	r=-0.483,<br>p=0.194	r=-0.517,<br>p=0.162	r=0.467,<br>p=0.213	r=0.433,<br>p=0.25	---	r=0.4,<br>p=0.291
**Ba**	r=0.833,<br>p=0.008 *	r=0.517,<br>p=0.162	r=0.917,<br>p=0.001 *	r=0.4,<br>p=0.291	r=0.433,<br>p=0.25	r=-0.417,<br>p=0.27	r=-0.483,<br>p=0.194	r=-0.433,<br>p=0.25	r=0.45,<br>p=0.23	r=0.383,<br>p=0.312	r=-0.133,<br>p=0.744	r=-0.883,<br>p=0.003 *	r=0.533,<br>p=0.148	r=0.917,<br>p=0.001 *	r=0.883,<br>p=0.003 *	r=0.4,<br>p=0.291	---

r - Spearman's correlation coefficient
* statistically significant ($p<0.05$)

Table S4. Spearman's correlation coefficient of elements in the bone of rats receiving nanogenistein.

	**Ca**	**Zn**	**K**	**Mg**	**Na**	**Fe**	**Cu**	**B**	**V**	**Cr**	**Mn**	**Ni**	**As**	**Se**	**Rb**	**Sr**	**Ba**
**Ca**	---	r=-0.433, p=0.25	r=-0.45, p=0.23	r=-0.85, p=0.006 *	r=0.533, p=0.148	r=0.95, p<0.001 *	r=-0.433, p=0.25	r=0.15, p=0.708	r=0.2, p=0.613	r=0.933, p=0.001 *	r=0.9, p=0.002 *	r=0.75, p=0.025 *	r=0.933, p=0.001 *	r=0.383, p=0.312	r=-0.35, p=0.359	r=0.917, p=0.001 *	r=0.917, p=0.001 *
**Zn**	r=-0.433, p=0.25	---	r=0.883, p=0.003 *	r=0.483, p=0.194	r=0.433, p=0.25	r=-0.433, p=0.25	r=0.967, p<0.001 *	r=0.483, p=0.194	r=0.533, p=0.148	r=-0.367, p=0.336	r=-0.417, p=0.27	r=-0.483, p=0.194	r=-0.417, p=0.27	r=0.4, p=0.291	r=0.883, p=0.003 *	r=-0.383, p=0.312	r=-0.433, p=0.25
**K**	r=-0.45, p=0.23	r=0.883, p=0.003 *	---	r=0.5, p=0.178	r=0.467, p=0.213	r=-0.45, p=0.23	r=0.883, p=0.003 *	r=0.6, p=0.097	r=0.45, p=0.23	r=-0.483, p=0.194	r=-0.5, p=0.178	r=-0.667, p=0.059	r=-0.433, p=0.25	r=0.417, p=0.27	r=0.8, p=0.014 *	r=-0.467, p=0.213	r=-0.517, p=0.162
**Mg**	r=-0.85, p=0.006 *	r=0.483, p=0.194	r=0.5, p=0.178	---	r=-0.433, p=0.25	r=-0.85, p=0.006 *	r=0.483, p=0.194	r=-0.2, p=0.613	r=-0.3, p=0.437	r=-0.883, p=0.003 *	r=-0.9, p=0.002 *	r=-0.833, p=0.008 *	r=-0.833, p=0.008 *	r=-0.533, p=0.148	r=0.35, p=0.359	r=-0.867, p=0.005 *	r=-0.917, p=0.001 *
**Na**	r=0.533, p=0.148	r=0.433, p=0.25	r=0.467, p=0.213	r=-0.433, p=0.25	---	r=0.483, p=0.194	r=0.433, p=0.25	r=0.767, p=0.021 *	r=0.717, p=0.037 *	r=0.45, p=0.23	r=0.417, p=0.27	r=0.15, p=0.708	r=0.467, p=0.213	r=0.867, p=0.005 *	r=0.467, p=0.213	r=0.433, p=0.25	r=0.45, p=0.23
**Fe**	r=0.95, p<0.001 *	r=-0.433, p=0.25	r=-0.45, p=0.23	r=-0.85, p=0.006 *	r=0.483, p=0.194	---	r=-0.383, p=0.312	r=0.15, p=0.708	r=0.283, p=0.463	r=0.933, p=0.001 *	r=0.95, p<0.001 *	r=0.833, p=0.008 *	r=0.983, p<0.001 *	r=0.433, p=0.25	r=-0.267, p=0.493	r=0.967, p<0.001 *	r=0.967, p<0.001 *
**Cu**	r=-0.433, p=0.25	r=0.967, p<0.001 *	r=0.883, p=0.003 *	r=0.483, p=0.194	r=0.433, p=0.25	r=-0.383, p=0.312	---	r=0.583, p=0.108	r=0.617, p=0.086	r=-0.367, p=0.336	r=-0.367, p=0.336	r=-0.45, p=0.23	r=-0.4, p=0.291	r=0.433, p=0.25	r=0.967, p<0.001 *	r=-0.35, p=0.359	r=-0.383, p=0.312
**B**	r=0.15, p=0.708	r=0.483, p=0.194	r=0.6, p=0.097	r=-0.2, p=0.613	r=0.767, p=0.021 *	r=0.15, p=0.708	r=0.583, p=0.108	---	r=0.85, p=0.006 *	r=0.117, p=0.776	r=0.2, p=0.613	r=-0.033, p=0.948	r=0.067, p=0.88	r=0.817, p=0.011 *	r=0.7, p=0.043 *	r=0.133, p=0.744	r=0.183, p=0.644
**V**	r=0.2, p=0.613	r=0.533, p=0.148	r=0.45, p=0.23	r=-0.3, p=0.437	r=0.717, p=0.037 *	r=0.283, p=0.463	r=0.617, p=0.086	r=0.85, p=0.006 *	---	r=0.283, p=0.463	r=0.383, p=0.312	r=0.283, p=0.463	r=0.233, p=0.552	r=0.883, p=0.003 *	r=0.75, p=0.025 *	r=0.317, p=0.41	r=0.367, p=0.336
**Cr**	r=0.933, p=0.001 *	r=-0.367, p=0.336	r=-0.483, p=0.194	r=-0.883, p=0.003 *	r=0.45, p=0.23	r=0.933, p=0.001 *	r=-0.367, p=0.336	r=0.117, p=0.776	r=0.283, p=0.463	---	r=0.967, p<0.001 *	r=0.883, p=0.003 *	r=0.917, p=0.001 *	r=0.417, p=0.27	r=-0.267, p=0.493	r=0.983, p<0.001 *	r=0.95, p<0.001 *
**Mn**	r=0.9, p=0.002 *	r=-0.417, p=0.27	r=-0.5, p=0.178	r=-0.9, p=0.002 *	r=0.417, p=0.27	r=0.95, p<0.001 *	r=-0.367, p=0.336	r=0.2, p=0.613	r=0.383, p=0.312	r=0.967, p<0.001 *	---	r=0.933, p=0.001 *	r=0.917, p=0.001 *	r=0.467, p=0.213	r=-0.217, p=0.581	r=0.983, p<0.001 *	r=0.983, p<0.001 *
**Ni**	r=0.75, p=0.025 *	r=-0.483, p=0.194	r=-0.667, p=0.059	r=-0.833, p=0.008 *	r=0.15, p=0.708	r=0.833, p=0.008 *	r=-0.45, p=0.23	r=-0.033, p=0.948	r=0.283, p=0.463	r=0.883, p=0.003 *	r=0.933, p=0.001 *	---	r=0.817, p=0.011 *	r=0.3, p=0.437	r=-0.3, p=0.437	r=0.9, p=0.002 *	r=0.917, p=0.001 *
**As**	r=0.933, p=0.001 *	r=-0.417, p=0.27	r=-0.433, p=0.25	r=-0.833, p=0.008 *	r=0.467, p=0.213	r=0.983, p<0.001 *	r=-0.4, p=0.291	r=0.067, p=0.88	r=0.233, p=0.552	r=0.917, p=0.001 *	r=0.917, p=0.001 *	r=0.817, p=0.011 *	---	r=0.417, p=0.27	r=-0.317, p=0.41	r=0.95, p<0.001 *	r=0.933, p=0.001 *
**Se**	r=0.383, p=0.312	r=0.4, p=0.291	r=0.417, p=0.27	r=-0.533, p=0.148	r=0.867, p=0.005 *	r=0.433, p=0.25	r=0.433, p=0.25	r=0.817, p=0.011 *	r=0.883, p=0.003 *	r=0.417, p=0.27	r=0.467, p=0.213	r=0.3, p=0.437	r=0.417, p=0.27	---	r=0.533, p=0.148	r=0.433, p=0.25	r=0.483, p=0.194

	Ca	Zn	K	Mg	Na	Fe	Cu	B	V	Cr	Mn	Ni	As	Se	Rb	Sr	Ba
**Rb**	r=-0.35, p=0.359	r=0.883, p=0.003 *	r=0.8, p=0.014 *	r=0.35, p=0.359	r=0.467, p=0.213	r=-0.267, p=0.493	r=0.967, p<0.001 *	r=0.7, p=0.043 *	r=0.75, p=0.025 *	r=-0.267, p=0.493	r=-0.217, p=0.581	r=-0.3, p=0.437	r=-0.317, p=0.41	r=0.533, p=0.148	---	r=-0.233, p=0.552	r=-0.233, p=0.552
**Sr**	r=0.917, p=0.001 *	r=-0.383, p=0.312	r=-0.467, p=0.213	r=-0.867, p=0.005 *	r=0.433, p=0.25	r=0.967, p<0.001 *	r=-0.35, p=0.359	r=0.133, p=0.744	r=0.317, p=0.41	r=0.983, p<0.001 *	r=0.983, p<0.001 *	r=0.9, p=0.002 *	r=0.95, p<0.001 *	r=0.433, p=0.25	r=-0.233, p=0.552	---	r=0.967, p<0.001 *
**Ba**	r=0.917, p=0.001 *	r=-0.433, p=0.25	r=-0.517, p=0.162	r=-0.917, p=0.001 *	r=0.45, p=0.23	r=0.967, p<0.001 *	r=-0.383, p=0.312	r=0.183, p=0.644	r=0.367, p=0.336	r=0.95, p<0.001 *	r=0.983, p<0.001 *	r=0.917, p=0.001 *	r=0.933, p=0.001 *	r=0.483, p=0.194	r=-0.233, p=0.552	r=0.967, p<0.001 *	---

r - Spearman's correlation coefficient
* statistically significant (p<0.05)
